# Liposome-Encapsulated Tobramycin and IDR-1018 Peptide Mediated Biofilm Disruption and Enhanced Antimicrobial Activity against *Pseudomonas aeruginosa*

**DOI:** 10.3390/pharmaceutics14050960

**Published:** 2022-04-28

**Authors:** Nouf M. Alzahrani, Rayan Y. Booq, Ahmad M. Aldossary, Abrar A. Bakr, Fahad A. Almughem, Ahmed J. Alfahad, Wijdan K. Alsharif, Somayah J. Jarallah, Waleed S. Alharbi, Samar A. Alsudir, Essam J. Alyamani, Essam A. Tawfik, Abdullah A. Alshehri

**Affiliations:** 1 National Center of Biotechnology, Life Science and Environment Research Institute, King Abdulaziz City for Science and Technology (KACST), Riyadh 11442, Saudi Arabia; nmalzahrani@kacst.edu.sa (N.M.A.); rbooq@kacst.edu.sa (R.Y.B.); aaldossary@kacst.edu.sa (A.M.A.); aabakr@kacst.edu.sa (A.A.B.); falmughem@kacst.edu.sa (F.A.A.); ajlfahad@kacst.edu.sa (A.J.A.); walsharif@kacst.edu.sa (W.K.A.); sjarallah@kacst.edu.sa (S.J.J.); salsadeer@kacst.edu.sa (S.A.A.); eyamani@kacst.edu.sa (E.J.A.); 2 Department of Pharmaceutics, Faculty of Pharmacy, King Abdulaziz University, Jeddah 21589, Saudi Arabia; wsmalharbi@kau.edu.sa

**Keywords:** cystic fibrosis, liposomes, tobramycin, innate defense regulator peptide-1018 (IDR-1018), biofilm, *Pseudomonas aeruginosa*, multidrug-resistant bacteria

## Abstract

The inadequate eradication of pulmonary infections and chronic inflammation are significant complications in cystic fibrosis (CF) patients, who usually suffer from persistent and frequent lung infections caused by several pathogens, particularly *Pseudomonas aeruginosa* (*P. aeruginosa*). The ability of pathogenic microbes to protect themselves from biofilms leads to the development of an innate immune response and antibiotic resistance. In the present work, a reference bacterial strain of *P. aeruginosa* (PA01) and a multidrug-resistant isolate (MDR 7067) were used to explore the microbial susceptibility to three antibiotics (ceftazidime, imipenem, and tobramycin) and an anti-biofilm peptide (IDR-1018 peptide) using the minimum inhibition concentration (MIC). The most effective antibiotic was then encapsulated into liposomal nanoparticles and the IDR-1018 peptide with antibacterial activity, and the ability to disrupt the produced biofilm against PA01 and MDR 7067 was assessed. The MIC evaluation of the tobramycin antibacterial activity showed an insignificant effect on the liposomes loaded with tobramycin and liposomes encapsulating tobramycin and IDR-1018 against both *P. aeruginosa* strains to free tobramycin. Nevertheless, the biofilm formation was significantly reduced (*p* < 0.05) at concentrations of ≥4 μg/mL and ≤32 μg/mL for PA01 and ≤32 μg/mL for MDR 7067 when loading tobramycin into liposomes, with or without the anti-biofilm peptide compared to the free antibiotic, empty liposomes, and IDR-1018-loaded liposomes. A tobramycin concentration of ≤256 µg/mL was safe when exposed to a lung carcinoma cell line upon its encapsulation into the liposomal formulation. Tobramycin-loaded liposomes could be a potential candidate for treating lung-infected animal models owing to the high therapeutic efficacy and safety profile of this system compared to the free administration of the antibiotic.

## 1. Introduction

Cystic fibrosis (CF) is a genetic disorder that affects mainly the lungs. It can occur due to the cystic fibrosis transmembrane regulator (CFTR) gene mutation, which controls the transmembrane flow of water and salts in pulmonary epithelial cells [1]. The mutation causes a defect in CFTR transport function, leading to the accumulation of thickened mucus that allows bacterial colonization and persistent lung infections [2]. Current CF treatments mainly focus on reducing inflammation, obstruction, or infection. The most common bacteria involved in CF patients’ lung infections is the Gram-negative bacterium *Pseudomonas aeruginosa* (*P. aeruginosa*), and most of those patients die from lung damage [3]. Several classes of antibiotics were reported to target *P. aeruginosa*, such as ceftazidime, imipenem, and tobramycin [4]. However, the difficulty in eradicating the bacterium from the site of infection, the low therapeutic efficacy of the currently used antibiotics, and the ability of bacteria to produce biofilms as an antimicrobial resistance could hinder the treatment of lung infections associated with CF [5].

Biofilm is a complex ensemble of microorganisms enclosed in an extracellular polymeric matrix consisting of polysaccharides, proteins, nucleic acids, and lipids [6]. The formation of biofilms is usually associated with the presence of severe infections that are difficult to eradicate and the development of antimicrobial resistance [7]. Due to their adaptive resistance to antibiotics, biofilms are difficult to treat [8]. Antimicrobial peptides (AMPs) are reported to have anti-biofilm activity and can act at different stages of biofilm formation with a different mechanism of action to be effective against a wide range of multidrug-resistant bacteria [9]. AMPs can disrupt the premature biofilm, downregulate the quorum sensing factors, inhibit biofilm formation and adhesion, and interact with intracellular targets, such as DNA, RNA, and proteins [10]. Such AMPs were reported to interfere with the bacterial cell signaling system via inhibiting the quorum sensing related genes that affect the biofilm formation in *P. aeruginosa*, hence reducing the bacterial adhesion on the surface [11]. Moreover, it has been demonstrated that targeting the polysaccharide intercellular adhesion (PIA) could modify the extracellular polymeric matrix architecture and therefore disrupt the bacterial biofilms [12]. An additional mechanism of anti-biofilm activity of AMPs is via reducing the expression of several genes that are involved in the transportation of binding proteins and biofilm formation, such as *icaA, icaD* and *icaR* genes, leading to the reduction in biofilm formation [13].

One of the strategies that can promote the anti-biofilm activity and enhance the antimicrobial efficacy of the applied antibiotics is anti-biofilm peptides, such as the innate defense regulator peptide-1018 (IDR-1018). This peptide is a synthetic derivative of bactenecin, a bovine host-defense peptide (HDP), which could facilitate the disruption of bacterial biofilm and thus increase the bacterial killing [14]. IDR-1018 is a cationic peptide due to the presence of four arginine residues. It was reported that IDR-1018 could cause a minimum effect on the membrane of the bacteria. Still, it might be translocated inside the bacterium by transforming its helical structure to β turn structure [14]. Additionally, it can prevent the lipopolysaccharide (LPS) membrane in Gram-negative bacteria by indirectly inducing pro-inflammatory cytokines [14]. IDR-1018 has very potent anti-biofilm activity. It was reported that this peptide might mediate the killing of several bacterial strains which produce biofilm, including the ESKAPE bacteria (*Enterococcus faecium*, *Staphylococcus aureus*, *Klebsiella pneumoniae*, *Acinetobacter baumannii*, *P. aeruginosa* and *Enterobacter species*) [15,16]. The potential features of the IDR-1018 peptide make it a promising strategy for bacterial infection management. The mechanism of IDR-1018 as an anti-biofilm is due to the binding and destruction of second messenger stress-induced nucleotides (p)ppGpp, which can promote the formation of biofilm [16].

The emergence of nanomedicine technology in drug delivery, such as dendrimers, liposomes, and polymeric nanoparticles, has markedly improved the therapeutic applications of different drugs, including antibiotics, to treat diverse diseases by increasing drug efficacy and reducing drug efficacy toxicity [17]. Several conventional medicinal drugs have low solubility, poor blood circulation time, and incompatibility with the biological tissues [18]. The loading of these therapeutics into a nanosized delivery system significantly impacts the bioavailability and biocompatibility profiles of the loaded drugs, owing to the novel physicochemical properties of these nanomaterials [19]. The encapsulation of antibiotics in liposomal nanoparticles can have a beneficial effect in terms of enhancing its stability and promoting the sustained release of the loaded antibiotic compared to the free antibiotic. The controlled release of the encapsulated antibiotics to the targeted site of action typically depends on the composition of the liposomal formulation [20,21]. This release method could avoid the premature release of antibiotics and reduce the frequency of the administered dose [22,23]. The delivery of antibiotics can also be improved through the conjugation of novel peptide-based therapeutics that could overcome different barriers to drug targeting, increase the biosafety profile of the selected drugs, and promote an antimicrobial activity against resistant microbial strains [24]. For example, it was reported that HDP can be designated as a prodrug by being conjugated directly to β-lactam antibiotics, such as cephalosporin, in which the designated prodrug can be cleaved by β-lactamase in the resistant bacteria to release the free active HDP [24,25].

One of the most successful nanosized delivery systems is liposomal nanoparticles, which can be used to encapsulate and transport antibiotics and anti-biofilm peptides to targeted infected tissues, which could enhance the therapeutic efficacy of antibiotics by facilitating the eradication of the bacterium and its biofilm [26]. Due to the phospholipid composition and the tuning of the physicochemical properties of lipid-based nanoparticles, such as fluidity, particle size, and zeta potential, the development of therapeutic-encapsulating liposomes as a localized therapy has grabbed massive attention in the past two decades [26,27].

Cationic liposomes exhibit a better affinity to and surface ionic interactions with the bacterial cell and biofilm [28,29]. In a comparative study by Drulis-Kawa et al., cationic liposomes encapsulating meropenem were found to be more effective against *P. aeruginosa* biofilms than anionic liposomes [30]. Liposomes have been favorably studied in lung diseases, since they could be developed into stably nebulized delivery systems and have the lipid bilayer structure that allows effective interactions with the cell walls [31]. Indeed, many liposomal aerosol-encapsulated antibiotics demonstrated remarkable effectiveness against CF and other lung infections [32,33]. For example, tobramycin is the most commonly used treatment for *P. aeruginosa* infections due to its ability to inhibit protein synthesis by binding to bacterial 30S and 50S ribosomal subunits. However, several bacterial species, including *P. aeruginosa*, developed resistance to this antibiotic which has urged scientists to chemically or physically conjugate tobramycin with other bioactive molecules such as dioctyl sulfosuccinate [34], bismuth–thiol [35], and clarithromycin [36] in liposomal formulations to increase its viability and effectiveness. These studies demonstrated that the new formulas were more efficient than the free tobramycin.

This study explores a potential therapeutic approach for lung infections by optimizing nanosized cationic liposomal formulations loaded with an antibiotic and an anti-biofilm peptide (IDR-1018) to evaluate their efficacy against PA01 and multidrug-resistant (MDR) 7067 strains of *P. aeruginosa*. This aim was achieved by initially considering the minimum inhibitory concentration (MIC) of different antibiotics against PA01 and MDR 7067 strains. Then, the most effective antibiotic was encapsulated into a cationic liposomal formulation in the presence or absence of IDR-1018 to be tested against the same *P. aeruginosa* strains.

## 2. Materials and Methods

### 2.1. Materials

Dulbecco’s Modified Eagle’s Medium (DMEM), ceftazidime, imipenem, and tobramycin were purchased from Sigma-Aldrich (Gillingham, UK), while the IDR-1018 was obtained from SYNTIDES (Shanghai, China). Cationic lipid 3β-[N-(N’, N’-dimethyl aminoethane)-carbamoyl] cholesterol hydrochloride (DC-Chol) and zwitterionic lipid 1, 2-dioleoyl-sn-glycero-3-phosphoethanolamine (DOPE) were bought from Avanti-polar lipids (Birmingham, UK). Human lung carcinoma cell line (A549) was obtained from the American type culture collection (ATCC): ATCC number—CCL-185. The MTS reagent (cell titer 96^®^ aqueous one solution cell proliferation assay) was supplied by Promega (Southampton, UK). Distilled water was generated through Milli Q (Millipore Corporation, Bedford, MA, USA).

### 2.2. Determination of the Antibacterial Minimum Inhibitory Concentration (MIC)

The antibacterial activity of ceftazidime, imipenem, and tobramycin antibiotics against two *P. aeruginosa* strains, a reference strain PA01 and a clinical isolate MDR 7067, were determined using the MIC assay. A serial dilution of the drugs (1024 to 0.5 μg/mL) in Mueller-Hinton broth was added to 96-well microtiter plates. Single pure colonies were collected from both strains to create bacterial suspensions (inoculum) using 0.5 McFarland standard, giving a cell density of 1.5 × 10^8^ colony-forming units (CFU)/mL measured by Kit DensiChek Plus Instrument (bioMérieux, Marcy L’Etoile, France) at 600 nm. Then, the bacterial suspensions were added to each well of the 96-well microtiter plates to achieve a final inoculum of 1 × 10^6^ CFU/mL. All 96-well microtiter plates were incubated overnight at 37 °C with a continuous shaking speed of 140 RPM. The endpoints of MIC were measured at absorbance 600 nm using a PowerWave XS2 plate reader (bioMérieux, Marcy L’Etoile, France). Positive and negative controls are wells containing only bacterium and culture medium, respectively, without adding any treatment. The results were evaluated according to the Clinical and Laboratory Standards Institute (CLSI) criteria for antimicrobial susceptibility testing [37]. Values are reported as the mean of three independent measurements ± standard deviation (SD).

### 2.3. Liposomal Nanoparticle Preparation

Liposome nanoparticles were formulated in the present work using the thin-film hydration method. The cationic lipid (DC-Chol) and the neutral lipid (DOPE) were dissolved initially in the methanol–chloroform mixture according to the manufacturer’s instructions. The two lipids were mixed in a round-bottom flask with a molar ratio of 1:1 to obtain a lipid concentration of 4 mM. Then, the organic solvent was evaporated by nitrogen gas until a thin film formed in the bottom of the flask. Next, the thin film was hydrated with 1 mL of tobramycin (5 mg/mL), IDR-1018 peptide (0.5 mg/mL), or a mixture of both antibiotic and anti-biofilm peptides. The final formulation ratios were 1:1 (*w*/*w*) for the total lipid and tobramycin; 1:0.1 (*w*/*w*) for the total lipid and IDR-1018; and 1:1:0.1 (*w*/*w*) for the total lipid, tobramycin, and the peptide. Control liposomes were prepared similarly but without the loaded antibiotic. Subsequently, the liposomes were passed through an extruder equipped with a 100 nm filter to reduce their particle size.

### 2.4. Determination of Encapsulation Efficiency (EE%) Using Ultra-High-Performance Liquid Chromatography-Tandem Mass Spectrometer (UHPLC-MS/MS)

Ultrafiltration centrifugation (Vivaspin^®^) tube was used to separate the free antibiotic from the liposome using a centrifugation speed of 4500× *g* for 1 h at 4 °C. The liposomal EE% was determined for formulations prepared with 1 mM total lipid. The filtrate was collected to determine the free antibiotic concentration. After that, 2 mL of deionized water was added to the filter, centrifuged at 4500× *g* for 20 min, then the filtrate was collected, and this step was repeated three times. Finally, 50% methanol was added to the filter and centrifuged at 4500× *g* for 15 min; then, the filtrate was collected. The quantification of the antibiotic in the collected filtrate samples was performed using a UHPLC-MS/MS.

All samples were centrifuged and, in some cases, diluted before analysis. The UHPLC system comprises a TCC-300RS Column compartment, WPS-300TRS autosampler, and an LPG-300RS quaternary pump with an integrated degasser. The identification and quantification of the antibiotic were conducted on a triple quadrupole mass spectrometer, TSQ Altis (ThermoFisher Scientific, Waltham, MA, USA). The ionization source was an electrospray ionization (ESI) operated at the following conditions: sheath gas 50, aux gas 10, gas temperature 300 °C, capillary voltage 3500 V. High purity (99.997%) argon gas was used for the collision cell. For UHPLC separation, a Thermo Scientific Syncronis C18 column (100 × 2.1 mm, 3 μm particle size) was used. The mobile phase was LC/MS grade containing water plus 0.1% formic acid (A) and methanol plus 0.1% formic acid (B), whereas the oven temperature was maintained at 40 °C. A linear gradient program was applied: 2.0 min 2% (B), 3 min from 2% to 98% (B), 1 min 98% (B), 1 min from 98% to 2% (B), 3 min 2% (B), and at a flow rate of 0.30 mL/min. Tobramycin was eluted at a retention time Rt = 0.59 min and quantified using ESI positive mode. The quantification of the antibiotic was attained using selected reaction monitoring (SRM) and the transition ions (m/z) 468→324 (14 eV), 468→205 (21 eV), and 468→163 (22 eV). Xcalibur software was used to process the acquired data, and a standard calibration curve with (R^2^ = 0.993) was plotted using a series of dilutions of tobramycin ranging from 1000 to 200 ppb. The *EE*% was calculated using the following equation:

(1) $$\mathrm{EE}\%=\left(C0-\frac{C1}{C0}\right)\times 100$$

where C1 is the concentration of free antibiotic and C0 is the initial antibiotic concentration.

### 2.5. Particle Size and Zeta Potential Measurement

Zetasizer Nano Series (Malvern Instruments, Malvern, UK) was used in the present work to measure the particle size and zeta potential of all prepared formulations. The measurements were conducted in distilled water at pH 7.4 and 25 °C. The values reported are the mean of three independent measurements ± SD.

### 2.6. In Vitro Cytotoxicity Assessment

The in vitro cytotoxicity assessment of liposomal formulations was performed using an MTS assay, according to [38]. The experiment was conducted against the A549 cell line to evaluate the cellular metabolic activity of the cells following the application of the investigated formulations. The living cellular models were used between passages 25–35. The culturing of human cells was routinely maintained in DMEM, supplemented with streptomycin 100 µg/mL, penicillin 100 U/mL, and 10% (*v*/*v*) fetal bovine serum (FBS). The cells were harvested using trypsin and counted with the trypan blue exclusion test, followed by seeding 1.5 × 10^4^ cells/well into 96-well plates. The cells were then incubated overnight in a cell culture incubator at 37 °C and 5% CO_2_. An amount of 100 µL of increasing concentration of the tested compound (from 1024 to 0.5 µg/mL) was then exposed to the human cells for 48 h. Cells incubated with 0.1% triton x-100 were used as a negative control, whereas cells incubated with DMEM only were used as a positive control. The consumed media were removed from the wells, and 100 µL of DMEM was added, followed by the addition of 20 µL of the MTS reagent into each well. The cells were then incubated for 3 h at 37 °C and 5% CO_2_. Cytation 3 absorbance microplate reader (BIOTEK instruments Inc., Winooski, VT, USA) was used following the incubation to measure formazan absorbance at 490 nm, and the cell viability% was calculated using the following equation:

(2) $$\mathrm{Cell}\;\mathrm{Viability}\%=\frac{\left(S-T\right)}{\left(H-T\right)}\times 100$$

where S is the absorbance of the cells treated with the applied formulations, T is the absorbance of the cells treated with triton x-100, and H is the absorbance of the cells treated with DMEM. Values reported are the mean of at least three independent measurements ± SD.

### 2.7. Anti-Biofilm Inhibition Assay

According to the previous studies, the biofilm formation was assessed by allowing the cells to adhere to the wells of 96-well microtiter plates [39,40]. From an overnight growth of the bacteria on LB agar, 200 μL of 10^6^ CFU/mL dilution in LB broth was inoculated into 96-well microtiter plates and incubated for 24 h at 37 °C. Then, the culture medium was replaced with fresh LB broth, and the plates were incubated for a further 24 h at 37 °C. After a 48 h incubation, the culture medium was aspirated. The adhered bacteria were treated using the following formulations: free antibiotic, liposomes loaded with the antibiotic, liposomes loaded with the antibiotic and the anti-biofilm peptide (IDR-1018), and empty liposomes. Positive and negative controls are wells containing only bacterium and culture medium, respectively, without adding any treatment. All the treatments were prepared in sterilized water with a serial dilution of the drugs (1024 to 0.5 μg/mL). The initial antibiotic concentration loaded into the liposomes (1024 μg/mL) was transferred into a new well, and certain volumes of sterilized water were added to dilute the original solution. The diluted samples were then used as a base solution to make further dilutions. The plates were incubated overnight at 37 °C; then, the MIC was measured at an absorbance of 600 nm using a PowerWave XS2 plate reader (bioMérieux, Marcy L’Etoile, France).

The plates were then washed three times with distilled water using BioTek ELx50 Microplate Strip Washer (BioTek, Winooski, VT, USA) to remove the bacterial suspension and single cells. An amount of 200 μL of a 0.1% crystal violet (CV) solution was applied to each well and then incubated at 25 °C for 15 min, followed by another washing cycle with distilled water. The plates were turned upside down to dry completely at 25 °C for approximately 30 min. To quantify the biofilm formation, 200 μL of 30% acetic acid was added to each well to dilute the CV-stained biofilm, and the plates were incubated at 25 °C for 15 min. The 200 μL of the solubilized CV-stained biofilm was transferred to new 96-well microtiter plates, and the absorbance was measured using 550 nm a PowerWave XS2 plate reader (BioTek, Winooski, VT, USA). The values reported are the mean of three independent measurements ± SD.

### 2.8. Statistical Analysis

Microsoft Excel 2019 software (Microsoft Corporation, Redmond, MA, USA) was used to calculate the mean and SD in the current work. A *t*-test was used to compare the results’ variance, in which *p* < 0.05 was considered statistically significant.

## 3. Results and Discussion

### 3.1. Determination of the Antibacterial Minimum Inhibitory Concentration (MIC)

Two bacterial strains of *P. aeruginosa* were used to evaluate the susceptibility to different concentrations of applied antibiotics: PA01 as a reference strain and MDR 7067 as a high resistance strain of *P. aeruginosa*. Table 1 summarizes the susceptibility test results of ceftazidime, imipenem, and tobramycin antibiotics tested against bacterial suspension (24 h) and biofilm-grown isolates (48 h). All tested antibiotics demonstrated anti-*P. aeruginosa* activity, but with a different bactericidal mechanism. Tobramycin is an aminoglycoside antibiotic that works by binding to the A-site on the 16S ribosomal RNA of the 30S ribosome, disrupting the bacterial protein synthesis [41]. Ceftazidime is a third-generation cephalosporin antibiotic that inhibits one or more essential “penicillin-binding proteins”, which may lead to an impaired cell wall homeostasis, interruption of bacterial cell wall formation, loss of cell integrity, and ultimately, bacterial death [42]. Imipenem is a broad-spectrum carbapenem antibiotic that interferes with PBP-2, PBP-1a, and PBP-1b proteins in *P. aeruginosa*, preventing the bacteria from adding a peptidoglycan polymer and therefore inhibiting the synthesis of bacterial cell wall which eventually leads to cell death [43].

The effect of antibacterial agents may vary against the same bacteria depending on whether the bacteria is in the planktonic or the biofilm stage. The results showed that tobramycin was the most effective antibiotic against the PA01 bacterium at both stages, the planktonic and after biofilm formation, compared to ceftazidime and imipenem, which required higher concentrations to inhibit this strain. The anti-biofilm activity of tobramycin might be due to the antibiotic diffusion through the biofilm matrix that may cause bacterial inhibition or adaption to the drug-stress responses [44]. The MDR 7067 isolate was more virulent against all antibiotics, and its biofilm exhibited higher resistance (>1024 μg/mL).

The findings of this study were consistent with the fact that the development of antibiotic-inactivating enzymes, which degrade or alter antibiotics, is a primary mechanism of intrinsic resistance in bacteria. Several antibiotics contain chemical bonds, such as esters and amides, that can be hydrolyzed by *P. aeruginosa* enzymes, including aminoglycoside-modifying enzymes and β-lactamases [45]. *P. aeruginosa* aminoglycoside nucleotidyltransferase (ANT) and aminoglycoside acetyltransferase (AAC) were identified to be involved in tobramycin inactivation by transferring an acetyl group to tobramycin’s amino groups or an adenylyl group to either the amino or hydroxyl group of tobramycin [46,47].

### 3.2. Determination of Tobramycin Encapsulation Efficiency% (EE%) Using Ultra-High-Performance Liquid Chromatography-Tandem Mass Spectrometer (UHPLC-MS/MS) 

There are several advantages to using liposomes as a delivery vehicle. One of them may be adjusted to the payload and release settings by using specific phospholipids and preparation techniques. Tobramycin was chosen as the most effective antibiotic against the two *P. aeruginosa* strains; therefore, it was loaded into the cationic liposomal nanoparticles and EE% and physiochemical properties were evaluated. High encapsulation efficiency (EE%) of tobramycin was achieved following the 8 µg/mL loading into the liposomal nanoparticles. The results showed an EE% of 94 ± 2% (≈7.5 ± 0.2 µg/mg), indicating that the antibiotic was successfully entrapped into the lipid bilayer membrane, which was determined by the developed UHPLC method, as shown in the Supplementary Materials section (Figure S1). This finding can promote the good quality of the prepared delivery system. It is essential to consider several factors to achieve an optimal liposome formulation with high drug encapsulation. These factors include the required vesicle size, the physicochemical properties of the substance to be encapsulated, polydispersity, and how easily and quickly the process can be scaled up [48].

### 3.3. Particle Size and Zeta Potential Measurement

The hydrodynamic diameters (i.e., particle size) and the nanoparticles’ surface charge (i.e., zeta potential) of the liposomal formulations were measured by the Zetasizer Nano Series instrument. The results are represented in Table 2. The particle size of all measured formulations following the passing through an extruder was below 200 nm, between 124 and 187 nm. The use of an extruder has a vital function in reducing prepared liposomal nanoparticle particle size by converting the MLV to ULV to achieve small particle size and low PDI measurements. The zeta potential values of the prepared liposomes were measured to assess the stability behavior of these liposomal formulations in the colloidal solution. The zeta potential of prepared cationic liposomes in the absence of tobramycin (empty liposomes) was 65.4 mV, while the zeta potential of the liposome-loaded tobramycin formulations with different concentrations ranged from 55.3 to 67.7 mV (Table 2). Increasing the tobramycin concentration showed no substantial effect on the prepared cationic liposomes’ particle size and zeta potential. Numerous previous research studies reveal that the size and charge of the liposome play a significant role in the overall efficiency of the designed preparation [49,50,51]. The liposomal formulations used in this study were cationic, which means they will have a higher affinity for negatively charged biofilms, reducing the time required to deliver antimicrobial agents to the infected site [50]. Additionally, the liposome formulations produced were designed to be smaller to improve their potential to penetrate the biofilm channels [49].

### 3.4. In Vitro Cytotoxicity Assessment

**Figure 1:**
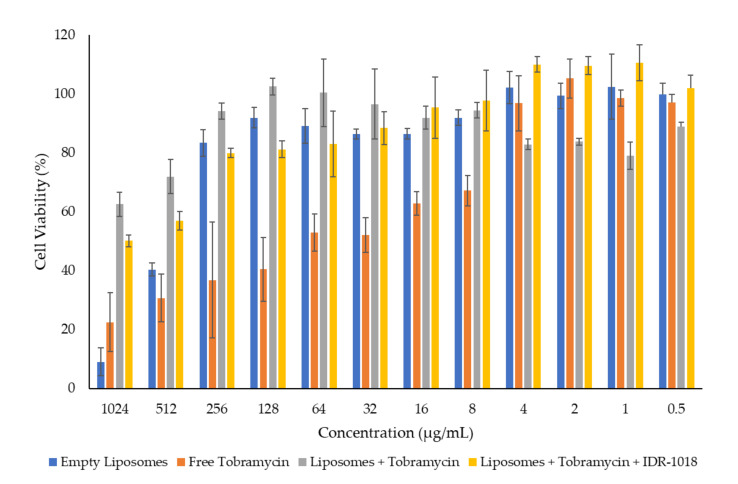
Cell viability of different concentrations of empty liposomes, free tobramycin, liposomes loaded with tobramycin, and liposomes loaded with both tobramycin and IDR-1018 peptide upon 48 h exposure with A549 cells. Results are represented as the mean ± SD (n = 3).

The in vitro cytotoxicity using MTS assay is vital for the biomedical application of nanoparticle formulations. It can define the optimal dose of the liposomes safe for further studies and, eventually, for clinical applications. In this experiment, increased concentrations of tobramycin (from 0.5 to 1024 µg/mL) encapsulated into the liposomes were evaluated against human pulmonary cancerous cells A549. Free tobramycin, empty liposomes, liposome-encapsulated tobramycin, and liposome-encapsulated tobramycin and IDR-1018 peptide were all assessed, as shown in Figure 1. The effect of different concentrations of the tested formulations on the cellular metabolic activity of A549 cells after 48 h cellular exposure exhibited that the free tobramycin has a dose-dependent cytotoxic effect. However, empty cationic liposomal formulation demonstrated low cellular viability (≈10%) of A549 cells at the highest concentration (1024 µg/mL). A lower concentration of empty liposomes, i.e., 256 µg/mL, showed higher cellular viability above 80% over 48 h, indicating the safety of using the liposomes at that concentration.

The encapsulation of tobramycin into the liposomes had a significant improvement (*p* < 0.05) in the cytotoxicity profile of the antibiotic. The cellular viability of tobramycin at a 256 µg/mL concentration was approximately 37%. In contrast, the cell viability of the tobramycin-loaded liposomes was about 94% at an equivalent concentration to the free antibiotic. No noticeable differences were shown in the cell viability of A549 cells with the tobramycin-loaded liposomes that contained the IDR-1018 peptide. This cytotoxicity study demonstrated that tobramycin is only safe at a ≤4 µg/mL concentration. Nonetheless, encapsulating tobramycin into the liposomal nanoparticle system has enhanced the safety profile of the antibiotic to reach ≤256 µg/mL.

### 3.5. Effect of Tobramycin-Loaded Liposomes against P. aeruginosa

**Figure 2:**
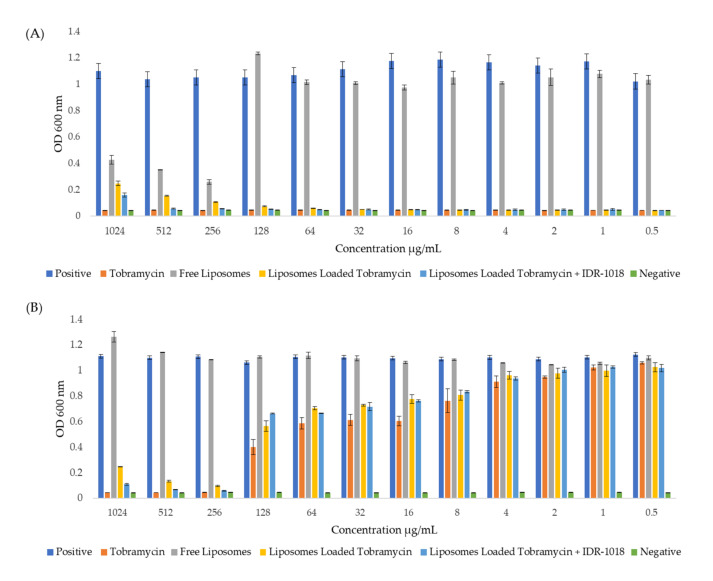
MIC results of different formulations against two *P. aeruginosa* strains after 24 h exposure. (**A**) The effect of the applied formulations on PA01 that was used as a control strain, in which it was significantly reduced with all tested tobramycin concentrations, while the MIC of the free liposomes was 256 μg/mL. (**B**) The effect of the applied formulations on MDR 7067 as a resistant strain showed that the free and formulated tobramycin had an MIC of 256 μg/mL, whereas there was no antibacterial activity for the empty liposomes. Results are represented as mean ± SD (n = 3). OD: optical density. Positive and negative controls are wells containing only bacterium and culture medium, respectively, without adding any treatment.

The MIC results of the free tobramycin, liposomes loaded with tobramycin, liposome loaded with both tobramycin and the IDR-1018 peptide, and empty liposomes against both *P. aeruginosa* strains, PA01 and MDR 7067, are shown in Figure 2. The free and loaded tobramycin formulations at all tested concentrations could inhibit the PA01 strain, while the MIC was increased to 256 μg/mL against MDR 7067. The empty liposomes did not affect the MDR *P. aeruginosa* isolate. Still, a slight antibacterial effect was exhibited at a ≥256 μg/mL concentration against the reference *P. aeruginosa* strain. This effect is not considered the MIC due to the remaining bacterial colonies. Finally, an insignificant difference was shown for the liposomes loaded with tobramycin and the IDR-1018 peptide in the bacterial growth of both *P. aeruginosa* strains compared to the tobramycin-loaded liposomes and the free antibiotic.

### 3.6. Effect of Tobramycin-Loaded Liposomes against Matured P. aeruginosa Biofilms

The two *P. aeruginosa* strains and their biofilms were grown planktonically and to a level of maturity (48 h), respectively. The biofilms were then treated with free tobramycin, liposomes loaded with tobramycin, liposome loaded with both tobramycin and the IDR-1018 peptide, and empty liposomes, and the results are presented in Table 3. The susceptibility of both *P. aeruginosa* strains in the planktonic state to tobramycin was reported to weaken after 48 h bacterial growth compared to 24 h. This might be due to the ability of developed biofilm to adapt to an applied antimicrobial agent and the emergence of biofilm tolerance over time. Tobramycin-free liposomal formulations showed no antibacterial effects for both bacterial strains after 24 and 48 h exposure at the highest tested concentration (i.e., 1024 μg/mL). The tobramycin-containing groups’ biofilm inhibitory concentrations (BICs) were 32 μg/mL and >1024 μg/mL for the reference and MDR strains, respectively, while the tobramycin-free systems showed no effect at all tested concentrations for both strains. The effect against the MDR 7067 isolate was >1024 μg/mL; however, this ratio remains too high and cannot be used medically.

### 3.7. Effect of Tobramycin-Loaded Liposomes against Biofilm Formation

**Figure 3:**
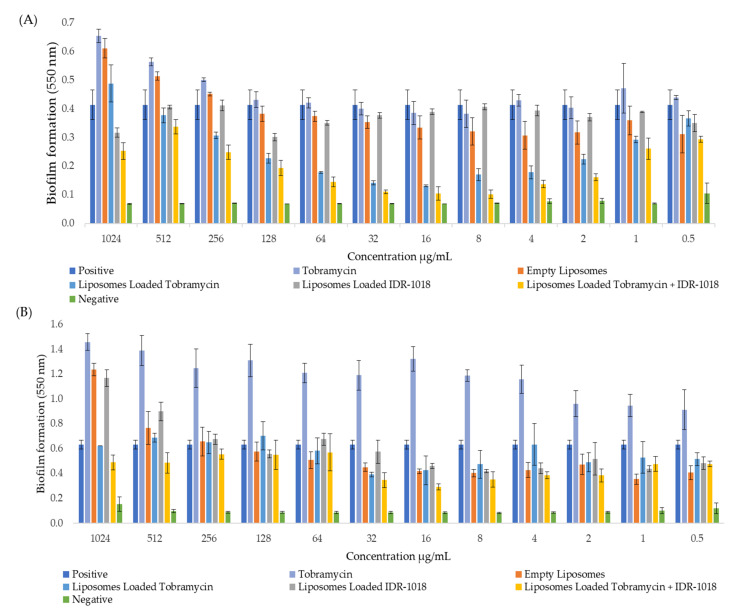
Biofilm formation of *P. aeruginosa* strains, showing biomass measurements, based on OD at 550 nm after 48 h exposure and the application of free tobramycin and different liposomal formulations. (**A**) PA01 as the control strain; and (**B**) MDR 7067 as the resistant strain. PA01 biofilm formation was reduced considerably with tobramycin-loaded liposomes and tobramycin and IDR-1018 peptide-loaded liposomes, with the activity being more significant (*p* < 0.05) than the free tobramycin. The increase in biofilm formation was observed gradually with the increasing tobramycin concentration. Results are represented as mean ± SD (n = 3). OD: optical density. Positive and negative controls are wells containing only bacterium and culture medium, respectively, without adding any treatment.

Biofilm formation was quantified using CV staining to identify the ability to adhere to polystyrene microtiter 96-well plates following 48 h bacterial exposure. An improvement in the impact of tobramycin was observed when liposomal nanoparticles were used as a carrier for this antibiotic. Both *P. aeruginosa* strains showed a significant decrease (*p* < 0.05) in the biofilm adherence at 4, 8, 16, and 32 μg/mL, following the treatment with tobramycin-loaded liposomes with or without the IDR-1018 peptide, as shown in Figure 3. The progressive increase in biofilm formation was observed gradually with increasing the concentration of tobramycin (Figure 3), probably due to the attempts of bacteria to protect their integrity via the formation of a self-produced matrix (biofilm) to raise the tolerance level towards the applied antimicrobial agent. Moreover, the liposomes loaded with anti-biofilm peptides insignificantly affected the PA01 strain. It followed the same anti-biofilm pattern as the tobramycin-loaded liposomes against the MDR 7067 isolate.

Bacterial biofilm has been shown to contribute to bacterial persistence and drug resistance, as the bacteria can tolerate different adverse conditions while in the dormant biofilm stage. It may occur either through the complex properties of biofilm structures or by the slow growth after the biofilm formation. In both cases, bacterial biofilm may substantially reduce the effect of antibacterial drugs [52]. Nevertheless, it was reported that decreasing the concentration of the antibacterial drug to a sub-lethal level could enhance the drug being diffused into the biofilm, consequently inhibiting the bacterial growth, which can survive the stress of the antibacterial agents [52].

The effect of antibacterial drugs may vary against the same bacterium, depending on whether the bacteria are in the biofilm or planktonic stage. In the case of the tobramycin treatment, it showed that this antibiotic was effective against grown bacteria in the planktonic stage but not in the biofilm stage. The low concentrations of tobramycin could not inhibit the bacteria after the biofilm formation through diffusing, indicating that this antibiotic has adapted to the drug-stress responses [44]. However, the encapsulation of the antibiotic in liposomes, with or without the anti-biofilm peptide, exhibited a reduction in the biofilm formation of the reference *P. aeruginosa* strain at concentrations of 4, 8, 16 and 32 μg/mL only, regardless of the addition of the anti-biofilm peptide.

The inhibition of bacteria by some antibiotics can stimulate biofilm formation by utilizing the release of bacterial cell components, such as polysaccharides, DNA, lipids, and proteins, which result from the disintegration of the bacterial cell wall. Consequently, the biofilm formation by the survived bacteria could be stimulated [53]. In addition, studies by Tahrioui et al. and Fricks-Lima et al. reported that the sub-MIC of tobramycin could increase the formation of *P. aeruginosa* biofilm [54,55]. The sub-MIC of antibiotics may stimulate the expression of biofilm formation related genes, such as QS related genes, and other virulence genes related to polysaccharides and alginate production [56,57,58,59]. In this study, the increase in biofilm formation in PA01 was observed after the concentration of 64 μg/mL gradually, and it is possibly due to the partial penetration of the antibiotic through the bacterial biofilm, while in the MDR bacterium, the formation of the biofilm was stimulated due to the sub-MIC of tobramycin. It was previously reported that the antibacterial effect of tobramycin might be related to its physicochemical properties, hence, enhancing the biofilm’s exposure time could help penetrate more across the biofilm even with high concentrations [60].

Several reports have studied the biofilm–antibiotic relationship [61,62]. A study showed that biofilm formation is adversely affected by the acquisition of quinolone by Gram-negative bacteria. In contrast, *P. aeruginosa* biofilm formation was enhanced considerably with the quinolone resistance trait [63]. Therefore, there are discrepancies among the results in this regard. The current study showed the effectiveness of tobramycin-loaded liposomes on PA01 and MDR 7067 bacteria. Furthermore, the biofilm production was significantly reduced (*p* < 0.05) at concentrations of ≥4 μg/mL and ≤32 μg/mL for PA01 and ≤32 μg/mL against MDR 7067, when loading tobramycin into liposomes, with or without the anti-biofilm peptide. The differences in the bacterial behavior towards the biofilm formation and antibiotics could be due to the bacterial adaption to the stress environment imposed by the presence of an antibacterial agent [52].

In addition, the anti-biofilm inhibitory effect of the empty liposomes might contribute to the cationic charge, which facilitated the electrostatic affinity between the liposomes and the anionic components of the biofilm, such as the proteins or the nucleic acid. A similar observation was shown in previous studies. Dong et al. studied the penetration of cationic liposomes into the cells of *S. aureus* and *P. aeruginosa*, and the results demonstrated that cationic liposomes adhered to the bacterial membranes better than the anionic liposomes, and the biofilm formation was suppressed after 5 min and 24 h exposure time. The study also concluded that by decreasing the liposomes’ size, improved penetration of the nanoparticles and inhibition of *P. aeruginosa* and *S. aureus* biofilms occurred [64]. Another study by Ibaraki et al. reported that the liposome properties could contribute to the antibacterial agents’ ability to act on the biofilms. The study showed that the anti-biofilm efficacy of the cationic PEG (i.e., polyethylene glycol) liposomes were enhanced by the cationic charge property of this nanoparticle system, and the PEGylation improved the permeability [65]. It was previously reported that the conjugation of a PEG moiety to the surface of nanoparticles has a crucial role in improving the efficacy of the loaded drug by reducing its clearance by the reticuloendothelial system (RES) or glomerular filtration [66]. The PEGylation of the administrated liposomes has significant impacts on decreasing immunogenicity and prolonging systemic circulation time [67]. These effects were due to the ability of the PEG moiety to protect the coated surface from interacting with blood components, hence avoiding the aggregation, opsonization, and phagocytosis of the nanoparticles [68]. Therefore, a PEGylated liposomal system could be suggested in further study, particularly when applying this formulated nanoparticle system in an animal model.

## 4. Conclusions

Patients with CF usually develop lung infections, particularly by *P. aeruginosa*, which could increase the mortality rate of those patients. The ability of bacteria to produce biofilm as antibiotic-resistant mechanisms can worsen CF’s treatment regimen and prognosis. Therefore, overcoming lung infection and biofilm formation may improve the life expectancy of patients who suffer from CF. This study aimed to promote the anti-biofilm effect of antibiotics and IDR-1018 through their encapsulation into novel liposomal nanoparticle formulation. This was proposed to mediate the disruption of the produced biofilm and improve the management of lung infections in CF patients as a potential therapy. A reference bacterial strain of *P. aeruginosa* (PA01) and a multidrug-resistant (MDR 7067) isolate were used. After determining the susceptibility to three antibiotics (ceftazidime, imipenem, and tobramycin), tobramycin was chosen to be loaded into the liposomal formulation along with the IDR-1018 peptide. The liposome formulations’ particle size and zeta potential were measured as <200 nm and >55 mV, while the EE% of the tobramycin-loaded liposomes was calculated as 94 ± 2%, indicating a successful preparatory criterion of these nanoparticle systems. These physicochemical properties of the liposomal systems could enhance the permeability and efficacy through the electrostatic interaction with the negatively charged bacterial biofilm. The safety profile of tobramycin against the lung carcinoma cell line was increased from a concentration of ≤4 µg/mL to ≤256 µg/mL upon encapsulating this antibiotic into the liposomal formulation. The antibacterial activity of tobramycin showed to be insignificant when being loaded with or without the IDR-1018 peptide. However, the biofilm formation was significantly reduced (*p* < 0.05) at concentrations of ≥4 μg/mL and ≤32 μg/mL for PA01 and ≤32 μg/mL for MDR 7067 when loading tobramycin into liposomes, with or without the anti-biofilm peptide compared to the free antibiotic, empty liposomes, and IDR-1018-loaded liposomes. The variances in the bacterial behavior to the formation of biofilm were probably due to the bacterial adaption to the stress environment imposed by the exposure of an antibacterial agent. These tobramycin-loaded nanoparticle systems could potentially be used to improve lung-infected animal models by enhancing the therapeutic efficacy and reducing the required effective dose of tobramycin as a step toward treating lung infections in CF patients. Nevertheless, understanding the role of the IDR-1018 peptide and other anti-biofilm peptides against lung infections should be further investigated, and an in vivo study should be undertaken to evaluate the safety and efficacy of such liposomal formulations in an infected animal model.

## Figures and Tables

**Table 1:** The MICs of three antibiotics against PA01 and MDR 7067 bacterial strains of *P. aeruginosa*.

Antibiotics	MIC (μg/mL)			
	PA01		MDR 7067	
	24 h	48 h	24 h	48 h
Ceftazidime	2	>512	128	>1024
Imipenem	8	32	512	>1024
Tobramycin	<0.5	8	256	>1024

**Table 2:** Particle size and zeta potential measurements of the prepared liposomes, with or without tobramycin.

The Concentration of Loaded Tobramycin (µg/mL)	Average Particle Size (nm)	Poly Dispersity Index	Zeta Potential (mV)
0 (empty liposomes)	124 ± 0.1	0.26 ± 0.13	65.4 ± 2.0
0.5	187 ± 0.2	0.39 ± 0.05	67.7 ± 0.6
1	124 ± 0.4	0.21 ± 0.02	55.3 ± 1.4
2	140 ± 0.7	0.27 ± 0.07	57.9 ± 0.4
4	127 ± 0.2	0.40 ± 0.01	62.1 ± 0.9
8	145 ± 0.8	0.31 ± 0.04	58.7 ± 3.2

**Table 3:** PA01 and MDR 7067 *P. aeruginosa* planktonic strains are susceptible after 24 h exposure (i.e., MIC) and 48 h exposure after biofilm-grown isolates (i.e., BIC) to free tobramycin, tobramycin-loaded liposomal formulations, empty liposomes, and peptide-loaded liposomes.

Formulations	MIC (μg/mL)			
	PA01		MDR 7067	
	24 h	48 h	24 h	48 h
Tobramycin	≤0.5	32	256	>1024
Liposomes + Tobramycin	≤0.5	32	256	>1024
Liposomes + Tobramycin + IDR-1018	≤0.5	32	256	>1024
Empty Liposomes	>1024	>1024	>1024	>1024
Liposomes + IDR-1018	>1024	>1024	>1024	>1024

## Data Availability

The authors confirm the data supporting the findings of this study.

## Supplementary Materials

The following supporting information can be downloaded at: https://www.mdpi.com/article/10.3390/pharmaceutics14050960/s1, Figure S1: The UHPLC calibration curve of tobramycin shows good linearity (R^2^ = 0.9928). The regression equation of tobramycin at a concentration range of 1000–200 ppb is y = 33295x−7 × 10^6^.
