# Supplementary material for: Liposome-Encapsulated Tobramycin and IDR-1018 Peptide Mediated Biofilm Disruption and Enhanced Antimicrobial Activity against Pseudomonas aeruginosa
Source: Pharmaceutics. 2022 Apr 28;14(5):960. doi: 10.3390/pharmaceutics14050960 (PMC9144307; doi:10.3390/pharmaceutics14050960)
Supplement: Supplementary file 1 [file pharmaceutics-14-00960-s001.zip › pharmaceutics-1671222-supplementary.pdf]

pharmaceutics

MDPI

Supplementary Materials: Liposome-Encapsulated Tobramycin and IDR-1018 Peptide Mediated Biofilm Disruption and Enhanced Antimicrobial Activity against *Pseudomonas aeruginosa*

Nouf M. Alzahrani [1], Rayan Y. Booq [1], Ahmad M. Aldossary [1], Abrar A. Bakr [1], Fahad A. Almughem [1], Ahmed J. Alfahad [1], Wijdan K. Alsharif [1], Somayah J. Jarallah [1], Waleed S. Alharbi [2], Samar A. Alsudir [1], Essam J. Alyamani [1], Essam A. Tawfik [1,*] and Abdullah A. Alshehri [1,*]

Tobramycin Determination and Quantification using Ultra-High-Performance Liquid Chromatography-Tandem Mass Spectrometer (UHPLC-MS/MS)

To quantify tobramycin in the drug-loaded liposomes, a UHPLC method was developed. Both the regression equation and R^2 for tobramycin were determined to be y = 33295x - 7E+06 and R^2 = 0.9928, respectively, indicating good linearity of this method at a concentration range of 1000-200 ppb.

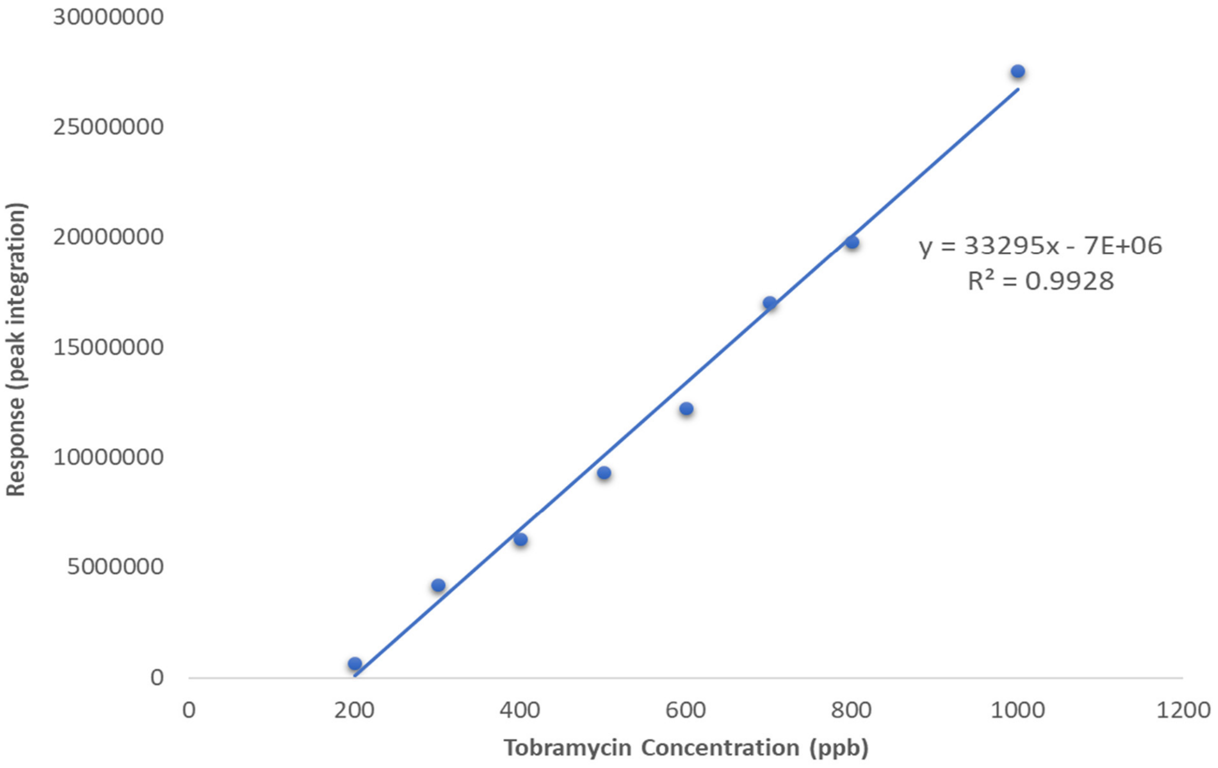

Figure S1. The UHPLC calibration curve of tobramycin shows good linearity (R^2 = 0.9928). The regression equation of tobramycin at a concentration range of 1000-200 ppb is y = 33295x - 7E+06.
